# Exploring the prevalence of gambling harm among active duty military personnel: a systematic scoping review

**DOI:** 10.1007/s10899-020-09951-4

**Published:** 2020-05-14

**Authors:** Marisa Paterson, Megan Whitty, Patrick Leslie

**Affiliations:** 1 Centre for Gambling Research, ANU Centre for Social Research and Methods, Research School of Social Sciences, ANU College of Arts and Social Sciences, Australian National University, Canberra, Australia; 2 Lecturer in Political Science at the School of Politics and International Relations, College of Arts and Social Sciences, Australian National University, Canberra, Australia

**Keywords:** Gambling, Military, Scoping review, Defence, Active duty

## Abstract

The prevalence of gambling harm among active duty military personnel is a largely unexplored topic. With different forms of social gambling often found within (or in close proximity to) military bases around the world, understanding the extent of gambling activities and consequent harms occurring within military contexts warrants further attention. This review aims to identify, describe and thematically synthesise published literature on gambling harm and related issues among active duty military personnel. Scoping review methods were applied in order to understand this relatively under-researched population and understand appropriate avenues for future research. A systematic multi-database text word search, incorporating search results from Scopus, Pubmed, Web of Science, PsychInfo, and the Journal Military Medicine, was conducted. A total of 11 sources met inclusion criteria, all originating from the United States of America. The results suggest a distinct gap in the current international literature on this topic. Despite gambling’s long and colourful association with defence downtime, research into gambling harm prevalence rates in relation to what could be considered a high-risk group is limited. Findings reveal that strategies to identify and address gambling harm within this population are severely lacking from the published literature and non-existent outside North America. Implications for understanding and addressing gambling harm among active duty personnel and directions for future research are discussed.

## Introduction

Wagering and games involving games of chance are widely accepted within Western defence cultures, as in broader society, as legitimate ‘recreational activities’. Gambling has historically been popular among defence members as a means to combat the stress, boredom, and isolation that can be experienced once deployed or upon returning home from active duty. These particular traits and past-times relating to gambling and culture are not unique to military contexts, as they are relevant in other similar sittings (i.e. mining, construction industries and prisons). These settings can be demographically skewed to young, male, high-risk-taking populations. In the United States, there is well-accepted support for gambling disorders^1^ (GD) to be recognized as a serious issue, affecting the health and wellbeing of a significant number of veteran and defence service members, and that it is not simply an innocuous pastime (Dighton et al. 2018; Whyte 2018). Indeed, the *National Defence Authorization Act* passed into United States (U.S.) federal law in 2018 mandated GD screening into the routine health checks of the U.S. Department of Defence (DoD).

The international research understanding gambling as an issue within the military has emerged predominantly from North America, and almost exclusively focuses on veterans (Biddle et al. 2005; Ellen L Edens and Rosenheck 2011; Kausch 2003). Research reporting the prevalence, socio-demographics, and psychiatric comorbidities of at-risk veterans, has focused particularly on the apparent link between GD and combat specific post-traumatic stress disorder (PTSD) (Biddle et al. 2005; Greden et al. 2010; O'Toole et al. 1998; Rosen et al. 2011). The overarching conclusion through the literature is that GD is underdiagnosed and undertreated among veterans (Drebing et al. 2001; Otto Kausch 2004; Westermeyer et al. 2005). How this corresponds to active duty populations is less well studied and will form the basis of this review.

This review is designed to systematically identify and describe all available research that addresses gambling within active-duty defence personnel. The primary objective is to examine the extent of evidence relating to gambling prevalence, GD and associated harm within active-duty Anglophonic defence settings (American, Canadian, U.K. or ANZAC) and to provide a systematic synthesis of the disparate sources. English-speaking defence settings were targeted because of the similar socio-cultural settings and understandings of gambling and gambling related harm.

## Method

A scoping review methodology was employed to capture the breadth of information available on the topic (Arksey and O'Malley 2005; Levace et al. 2010). Arksey and O’Malley’s five-stage approach for conducting a scoping review guided this research.^2^ For quality and transparency this review also adhered the Preferred Reporting Items for Systematic Reviews and Meta-Analyses (PRISMA) guidelines and includes a PRISMA flow diagram of search results and study selection (Moher et al. 2010). In line with scoping review methods assessment of the quality of the studies was not undertaken.

Ethics approval was not sought, as the review did not contain any studies with human participants performed by any of the authors.

### Search strategy

The following databases were searched to identify eligible sources up to and including December 2018: Scopus, Pubmed, Web of Science, PsychInfo, and the Journal Military Medicine (using Web of Science’s search tool). Details of the Boolean searches applied in each database are described in Table 1. To supplement the references captured from the formal database searches, a search of the grey literature was conducted. Manual searches of Google and Google Scholar were conducted and reference lists of eligible full texts were screened for potentially relevant articles.

**Table 1:** Database search terms and results

Database	Query	Number of studies
Scopus	(gambling AND military) OR (gambling AND "servicemen" OR "service people") OR (gambling AND "service personnel") OR (gambling AND army) OR (gambling AND navy) OR (gambling AND "air force") OR (gambling AND marines) OR (gambling AND anzac)	98
Pubmed	(gambling AND military) OR (gambling AND "servicemen" OR "service people") OR (gambling AND "service personnel") OR (gambling AND army) OR (gambling AND navy) OR (gambling AND "air force") OR (gambling AND marines) OR (gambling AND anzac)	55
Web of science	(gambling AND military) OR (gambling AND "servicemen" OR "service people") OR (gambling AND "service personnel") OR (gambling AND army) OR (gambling AND navy) OR (gambling AND "air force") OR (gambling AND marines) OR (gambling AND anzac)	144
PsychInfo	Any Field: gambling AND Any Field: military OR Any Field: navy OR Any Field: air force OR Any Field: marines OR Any Field: anzac OR Any Field: servicemen OR Any Field: service people OR Any Field: army OR Any Field: service personnel	15
Military Medicine (using WoS)	TOPIC: (gambling) AND PUBLICATION NAME: ("Military Medicine")	5
Sum		317

### Source selection

To be considered eligible for inclusion, sources had to: focus on gambling prevalence; have active-duty military personnel from English speaking geographic regions (namely the U.K., Australia, New Zealand, Canada, or the U.S.) as the target population, and; be available online in full text. No restrictions were placed on study design or publication dates and no preference was given to qualitative or quantitative methodology. Foreign-language material was excluded because of the parameters of the focus of the review and potential translation costs. Therefore, sources were included if they were primary studies (i.e. involving the collection of original primary data through directly measuring the outcome of interest within the relevant population), secondary studies involving the analysis and interpretation of primary research, or discussion papers.

**Fig. 1:**
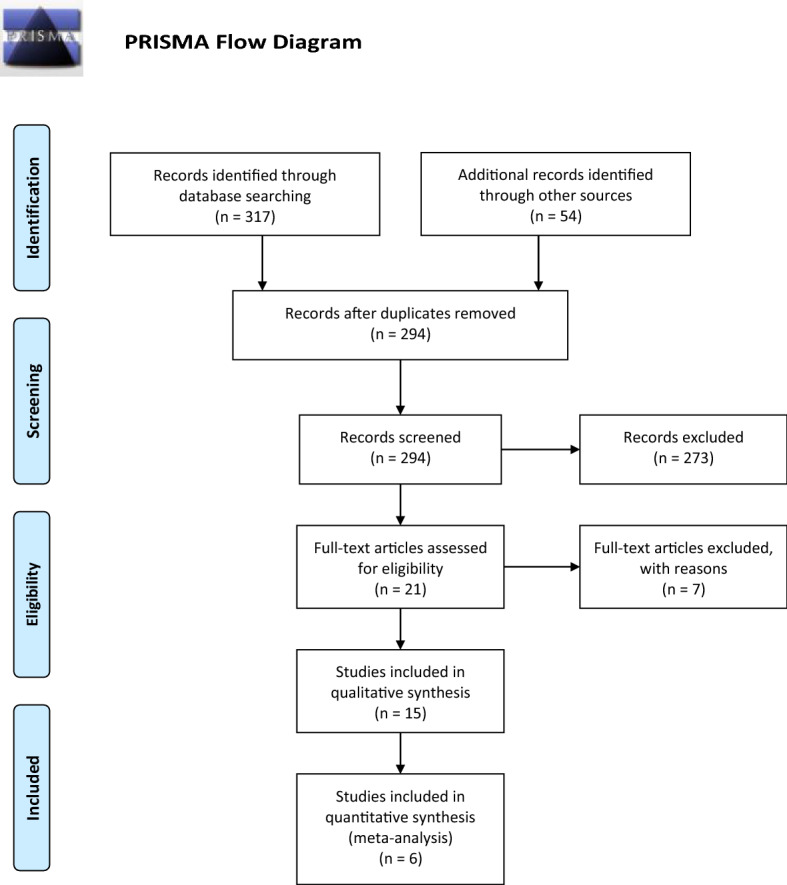
PRISMA statement

After the initial database search, duplicates were removed. The screening of titles and abstracts, and the selection of articles from retrieved potentially relevant full manuscripts, were conducted by two reviewers (PL and MW) using the selection criteria described above. The reviewers independently classified the articles as ‘include’, ‘unclear’ or ‘exclude’, with discrepancies being resolved by discussion or referral to a third reviewer (MP). Full manuscripts that did not fulfil all of the criteria were excluded, with reasons for their exclusion documented. See Fig. 1 for the PRISMA flow diagram outlining the search and selection process.

### Data extraction

Two reviewers independently conducted the data extraction. The data extraction form used was adapted from the ‘Data collection form for intervention review – RCTs and non-RCTs’ of The Cochrane Collaboration^3^ (see “Appendix”). Extraction items included publication details (authors and date), source setting, participants, research design and objectives, sample size, results and outcome measures (if applicable), and key author conclusions/recommendation.

### Analysis

Thematic synthesis was chosen to analyse these data collectively. Therefore, data analysis was undertaken in three stages: (1) evidence mapping, (2) identification of evidence gaps and (3) synthesis of selected research areas. In other words, iterative coding was applied to the extracted data which was organized into descriptive themes that were then used to generate broader analytical discussion.

## Results

### Descriptive characteristics of reviewed sources

As outlined, search terms were intentionally broad to capture as much relevant literature as possible, yielding 317 references for further processing. Our informal search strategy yielded 54 studies for further review. After accounting for duplicates our search of online databases yielded 294 unique references (See Fig. 1). The final sample (n = 11) of sources all originated from the U.S. and included four journal articles (Kennedy et al. 2006; Little and Hecker 1988; Steenbergh et al. 2008; Weis and Manos 2007) and one online article (Ashley and Shannon 2017), two Health Survey Reports (), a book chapter (Kennedy et al. 2006), a report (GAO 2017), an online bulletin (GSU 2010) and a conference presentation (poster) (Wilson et al. 2018). Eligible sources were exclusively from the U.S. and mainly from a quantitative operational research perspective. A few of the selected sources looked at specific populations—residents of a Military Base in Okinawa, Japan (Kennedy et al. 2006), a sample of U.S. Air force (USAF) recruits in their second week of basic training (Steenbergh et al. 2008), and outpatients of a Naval medical centre in Portsmouth, Virginia, USA (Weis and Manos 2007).

Qualitative or text-based sources were analysed separately. Tables 2 and 3 present the characteristics of included sources by study design, i.e. quantitative studies are analysed (Table 2) separate to the text-based research (Table 3).

**Table 2:** Summary of quantitative data from included studies

Author/year	Type	Sample size	Participants	Location	Gambling screen/result
Bray (1992 )	Health survey report	16,935	Randomly sampled active military personnel in the US Military	USA	Problem gambling (DSM-III gambling disorder) lifetime prevalence
					7.1% report 1 or more gambling problems;
					2.0% report 3 or more gambling problems;
Bray (2002)	Health survey report	12,756	Randomly sampled active military personnel in the US Military	USA	Prevalence of PG DSM-IV gambling disorder, lifetime Prevalence
					6.3% report 1 or more gambling problems;
					2.3% report 3 or more gambling problems;
					1.2% report 5 or more gambling problems (suggestive PG)
Kennedy et al. (2006)	Journal article	35	US Military personnel seeking gambling counselling in the first year of a gambling treatment in a US Naval Hospital	Okinawa, Japan	South Oaks Gambling Screen
					Mean score: 10.53 (SD 4.16)
Steenbergh (2008)	Journal article	31,104	Sample of U.S. Air Force recruits	USA	Custom abbreviated 12-month gambling screening questionnaire:
					Level 2 gambling at 6%
					Level 3 gambling at 1.9%
Weis and Manos (2007)	Journal article	584	Outpatients presenting to a Naval Medical Center psychiatry clinic	Portsmouth, VA, USA	South Oaks Gambling Screen:
					Mean score: 0.34 (SD = 1.10)
Wilson et al. (2018)	Poster presentation	861	Data from active duty personnel in the U.S. Armed Forces who had been diagnosed with Pathological gambling (ICD 9 312.31)	USA	NA

In the Steenbergh (2008) study, Level 1 gambling is defined as ‘recreational’ and people in this category “typically experience little or no resulting financial, psychological or interpersonal harm” (p. 452). Level 2 gamblers “usually experience some gambling-related symptoms or problems, but do not meet diagnostic criteria for pathological gambling disorder” (p. 452). Finally, level 3 gamblers “present with chronic & debilitating problems, significant impairment in daily functioning, and loss of control over their gambling” (p. 452).

Mean and standard deviation recovered from Table 1 of Weis and Manos (2007) using a weighted average of military personnel (excluding dependents)

Since all personnel in this study had already been diagnosed with a gambling disorder, no measure of a gambling screen was reported. The study detailed the demographic characteristics of this sample.

**Table 3:** Text based summary of included studies

Author/year	Type	Study aims	Method	Results and recommendations*
Ashley and Shannon (2017)	Online article	Policy and research activity overview	Literature review of GD prevalence and gambling related harm in US context	Problem Gambling can be a significant co-occurring disorder and should be included in Military Health coverage. All assessments for Addiction and Mental Health issues with Military Personnel and Veterans should require assessing for GD. More research is needed relative to the incidence of GD in the Military
GAO (2017)	Performance audit report	Investigate prevalence, assesses approaches to screening, diagnosis & treatment; and evaluate DOD/CG guidance to addressing GD	Audit of Military Health System (MHS) Data Repository, and 2 × literature searches re GD prevalence in the general population and military personal	Less than 0.03 percent of the average number of service members in each year—were diagnosed with GD or were seen for problem gambling in fiscal years 2011—2015 MHS, reflecting a tiny proportion of the estimated prevalence of problem gambling from the general and military process. Voluntary help seeking is low
GSU (2010)	Online resource	Overview of GD prevalence and gambling related harm in the Military & Veteran populations	Literature and policy activity review in the US context	Need to screen military personnel and veterans for PG is evident. Screening for GD provides opportunities to intervene, which may reduce prevalence and alleviate associated negative impacts to public health
Kennedy (2016)	Book chapter	Literature and policy review of GD in the US military compared to comorbid conditions	Literature review and policy recommendations	The frequency of suicidality and other comorbid mental health issues and substance use disorders means that screening for and evaluation of GD is not as simple as a preliminary substance abuse evaluation, for example. Tailoring of individual and group therapy are important considerations for GD treatment options, as is the different counselling services that may be necessary to provide on a case-to-case basis (financial or marital counselling, spousal education, emergent suicide risk assessment)
NCPG (2007)	Policy paper	To review the recent research and policy with the intention of drawing conclusions and directing future policy	Review of past policy and research, policy critique	Current approaches seem to be punitive rather than therapeutic. There is a need for independent research by specialists into gambling and GD in the military. Ongoing data collection efforts must be maintained and improved. In addition to the inclusion of gambling questions in the Worldwide Survey of Health Behaviours, services need to develop clear policy around the enforcement of gambling rules and regulations

DODDepartment of defence, GDGambling disorder, MHSMilitary Health System, USUnited States

*Key findings that relate to the scoping review question

**Table 4:** Thematic mapping

		Ashley and Shannon (2017)	Bray (1992, 2003)	GAO (2017)	GSU (2010)	Kennedy et al. (2006)	Kennedy (2006)	Little (1988)	Steenbergh (2008)	Weis and Manos (2007)	Wilson et al. (2018)
Themes	Subthemes										
Defence culture	Stigma**	Y	N	Y	Y	Y	N	N	N	Y	N
	Barriers to treatment options**	Y	N	Y	N	Y	N	N	N	Y	N
	Suspected underdiagnoses*	N	Y	Y	N	N	N	N	N	Y	N
	Screening (or lack of)***	Y	N	Y	Y	N	Y	N	N	Y	Y
	Impact on military readiness***	Y	Y	Y	Y	N	N	Y	Y	N	N
	Punitive response to GD***	Y	N	Y	Y	Y	Y	Y	N	N	N
Risk factors	Inadequate guidelines/policy**	N	N	Y	N	Y	Y	Y	N	N	Y
	Risk relative to the general population***	Y	Y	Y	Y	N	N	N	Y	Y	Y
	Demographic variables***	N	Y	N	Y	Y	N	N	Y	Y	Y
	Availability of gambling activities (on/off line)***	Y	N	N	Y	Y	N	N	Y	Y	Y
	Assessment of prevalence**	N	Y	Y	Y	N	N	N	Y	Y	N
	Risk-taking/sensation seeking*	Y	N	N	N	N	N	N	Y	N	N
Comorbidity	Mental health & PTSD**	N	N	Y	Y	Y	N	N	N	Y	Y
	Alcohol and substance misuse***	N	Y	Y	Y	Y	Y	N	Y	Y	Y
	Suicide prevention**	Y	N	Y	Y	Y	N	N	N	Y	N
	Secondary diagnosis/behavioural disorder**	N	N	Y	Y	Y	N	N	Y	N	N
	Combined screening/treatment*	N	N	Y	N	Y	Y	N	N	N	N
Key											
*	Minor theme (< 3 'Y's)	3									
**	Midrange theme (4, 5 'Y's)	7									
***	Majar theme (6 > 'Y's)	7									

Themes	Subthemes	Quote
Defence culture	Stigma	
	Barriers to treatment options	
	Suspected underdiagnoses	
	Screening (or lack of)	Without incorporating medical screening questions specific to [GD], gambling problems may not be identified until they reach a critical point affecting the individual’s readiness in addition to harming the financial situation of the servicemember ….” GAO 2017
	Impact on military readiness	
	Punitive response to GD	
	Inadequate guidelines/policy	
Risk factors	Demographic variables	
	Risk-taking/sensation seeking	
	Availability of gambling activities (on/off line)	Given the ease of accessibility across jurisdictional boundaries, it may be an enticing entertainment option for those posted overseas or in geographically isolated areas (Ashley)
	Risk relative to the general population	
	Assessment of prevalence	
Comorbidity	Veterans at increased risk	
	Mental health & PTSD	
	Alcohol and substance misuse	If personnel are not screened for gambling-related problems when they enter alcohol treatment, these problems may very well go undetected. Furthermore, an even higher prevalence of gambling-related problems might be found among those personnel whose alcohol problems are currently undetected or untreated. Overall, these data support the relationship found in existing studies between alcohol use and abuse and gambling-related problems. Finally, given that veterans have been found to have problems with pathological gambling and alcohol use (Daghestani et al., 1996), it is not surprising to find a similar situation among active-duty personne (Bray 2003)
	Suicide prevention	
	Secondary diagnosis/behavioural disorder	“[G]ambling is significantly different from substance abuse in relation to military policy and confidentiality. Whereas substance abuse has to be reported to a command, a gambling problem per se does not... Unless a service member who seeks help for pathological gambling presents with suicidality or another issue that requires mandatory reporting, he or she will enjoy a significant degree of confidentiality and can self-refer.” Kenedy 2016
	Combined screening/treatment	

Examination of the extracted data revealed three key concepts spanning the literature (represented by the included studies) represented by the umbrella terms; ‘military culture’, ‘risk factors’ and ‘comorbidity’. Major themes (n = 6 +), mid-range (n = 4–5), and minor themes (n = 3–4) were organized into their associated category and tabulated in Table 4. To ensure attention was weighted based on the prominence of the themes themselves (and not imposed on the data by the review team) the frequency with which themes occurred determined its status as a major, mid, or minor theme.

#### Key concept 1—military culture

Features unique to military environments may inadvertently encourage engagement with gambling activities and at the same time contribute to concealment of potential issues or deterring access to treatment. Review findings highlight several related issues.

#### Confidentiality and punitive responses to gambling disorder

The hierarchical (chain-of-command) structure of military forces is problematic in that supervisors are promoted as a primary resource that can provide advice, referral and support, however these authority figures also have the power to discipline, demote, and even discharge personnel with GD related issues. Soldiers in a Naval medical centre in the U.S. reported failing to disclose problematic gambling behaviour due to shame and confusion about the military’s confidentiality policies (GSU 2010; Kennedy et al. 2006). Other studies also found that GD can affect the ‘financial and psychological well-being of military personnel and, thus, in turn, can have a negative effect on military readiness’ (Bray et al. 2002). Again, another reason why military personnel are reluctant to disclose such issues.

Further to this, studies suggest that the military is quick to reprimand people with GD while being slow to offer meaningful assistance, arguing that defence culture has little tolerance for behavioural problems. Clearly, the risk of prosecution for those who had committed crimes related to their gambling could discourage those with GD from seeking treatment and appropriate counselling. Thirty years ago, Little and Hecker (1988) examined the use of pathological gambling in the context of ‘insanity’ pleas. Their discussion of pathological gambling in relation to the changing guidelines that dictate military criminal trials concludes by urging for greater acceptance of such lines of defence in military courts (Little and Hecker 1988). Two decades later the military approach still appears focused on treating problems associated with GD as punishable offenses with minimal understanding, or regard for the underlying treatable disorder of the individual (NCPG 2007). However, Kennedy et al., report that of the 25 active-duty members referred for treatment in their 2006 study, 21 were retained in the military, whereas four were court-martialled and subsequently discharged (Kennedy et al. 2006).

#### Screening for gambling disorder

Many sources in this review state or otherwise imply that GD diagnosis is not common in defence populations, echoing the broader literature (Drebing et al. 2001). Inadequate screening for GD amongst active duty personnel may be the reason for it being underdiagnosed (Ashley and Shannon 2017; GAO 2017; GSU 2010; NCPG 2007). Implementing systematic screening that specifically targets gambling behaviour provides *opportunities to intervene when appropriate. Such interventions may well reduce the incidence and prevalence of GD, as well as alleviate associated negative impacts to public health (*Korn and Reynolds 2009*).* Furthermore, confidential ways of seeking treatment should be put in place to address barriers to self-referral. Raising awareness of gambling harm would also assist the implementation of effective screening and referral processes.

The U.S. Government Accountability Office (GAO) released a report that examined GD in the military and recommended that questions specific to gambling be incorporated as part of DoD’s screening process (GAO 2017). DoD agreed to update processes and treat gambling as an addiction but initially declined to adopt screening recommendations. In 2018, the *Gambling Addiction Prevention Act* was introduced requiring DoD to screen and survey service members for gambling disorders, as well as a new provision calling for the development of policies and programs to prevent and treat GD. Legislating screening, treatment and understanding GD prevalence is unique to the US.

#### Key concept 2—risk factors

The second overarching theme relates to the numerous risk factors for GD among military personnel relative to the general population.

#### Availability of gambling

Recent studies show gambling is increasingly available and impactful in military life (Whiting 2016; Whyte 2018; Wilson et al. 2018). As in the broader research on GD in the general population, accessibility of gambling activities is a major topic of interest.

Ashley (2017) claim that slot machine gambling is available on most oversees US military bases (i.e. excluding bases located in the US), a form of gambling most often associated with GD. Wilson et al. (2018) note that higher rates of GD diagnosis occur in bases outside the continental United States. Kennedy et al. (2006) argues that, considering the increased availability of gambling products, mental health and addiction programs in overseas locations often do not provide services commensurate with the level of risk that gambling presents.

There is also the potential need to assess the impact of online gambling on military personnel (Ashley and Shannon 2017). In Australia, it is not permitted to use defence internet and communication technologies (ICT) or resources to access, or download from, gaming sites (i.e. sites that enable gambling/risking money or anything of value). Although with the proliferation of online gambling apps and the use of mobile phone and other technologies, the impact of such restrictions may be minimal and is currently unknown.

#### Demographics

The review found that studies of gambling in the military typically expect active service members to be at an increased risk of GD (Bray et al. 1992; GAO 2017; GSU 2010; Kennedy et al. 2006). Prior studies of the general population in the U.S. note that the increased rates of GD among males, people of colour and people under 30 years of age suggest a higher risk rate of GD among active duty personnel (see also Weis (2007), GSU (2010) and Steenbergh et al. (2008). Weis and Manos (2007) make the point that there is higher general prevalence among young males and the defence forces are largely made up of people fitting this demographic. A profile of the active-duty pathological gambler is offered by Kennedy et al. (2006) after the first year of the Okinawa program: the majority being self-referring males, with a mean age of 33.2 years (Kennedy 2006). Weis’ (2007) study of rates of GD prevalence at a naval psychiatry clinic showed that men and active duty personnel (relative to the family member’s also accessing services at the clinic) were at an increased risk of scoring highly on the South Oaks Gambling Screen (SOGS). Steenbergh et al. (2008) published associations between demographic characteristics and GD in the USAF. The findings indicated that the risk of pathological gambling was significantly increased among males and ethnic minorities (Steenbergh et al. 2008).

#### Assessment of prevalence

Three sources in the review were studies of GD prevalence; two commissioned by the US DoD for the entire US military and another among patients at a naval psychiatric facility.

A feature common to the prevalence studies was the lack of comparable measures or populations when estimating prevalence. Weis (2007) used SOGS and Bray et al. (1992, Bray et al. 2002) used the DSM-III and DSM-IV Screens for Gambling Problems respectively. The differences in population and measurement tools makes Weis (2007) difficult to compare with the general military population. However, a mean SOGS score of 0.34 (SD = 1.10) among serving military personnel in a psychiatric treatment facility (not presenting with GD initially) is suggestive of significantly increased risk relative to the general population (Weis and Manos 2007).

The US DoD conducted two studies assessing the lifetime prevalence of GD (Bray et al. 1992, 2002). Around two percent of respondents reported experiencing three or more gambling related problems on the DSM checklist, suggestive of problem gambling. Pathological gambling (defined as reporting 5 or more gambling related problems in the DSM-IV Screen for Gambling Problems) was measured at 1.2%, a figure that was seen as roughly comparable to the general US population, estimated by a meta study to be 1.5%^4^ in 1999.^5^

#### Key concept 3—comorbidity

The literature has consistently reported that GD can be a significant co-occurring disorder and there is evidence of an association between disordered gambling and various comorbid psychiatric and substance use conditions (Hartmann and Blaszczynski 2018). Discussed frequently by both health professionals and reporters, military personnel deal with serious consequences of substance abuse, mental health problems and suicide (Ellen L. Edens 2012; Holmes et al. 1998; Iversen et al. 2011; Kausch 2003; Langston et al. 2010). Ashley and Shannon (2017) suggests that it is crucial to recognize that comorbidity with other diagnosis is important to understanding how/why gambling may become problematic in military personnel.

#### Risk taking

Ashley (2017) notes that military personnel have higher rates of risk taking and sensation seeking than their civilian counterparts. Studies of the general population suggest that these variables correlate highly with episodes of GD, though we found no evidence specifically linking general risk-taking and sensation seeking with GD. However, GD and specifically *health-risk* behaviour in the U.S. Air Force (Steenbergh et al. 2008) does appear to be correlated. Survey measures of health risks common in the military such as reckless driving, physical fighting, cigarette smoking, binge drinking, and others were shown to have positive correlations with gambling participation (Whiting 2016). While physical fighting and riding with an intoxicated driver were associated with more serious forms of gambling disorder (Steenbergh et al. 2008).

#### Alcohol and substance misuse

Eight out of eleven studies in this review also acknowledge the potential for gambling issues to be more likely in personnel who have also reported alcohol and substance misuse problems. Bray (1992, 2002) found that, as alcohol consumption rose, so too did the prevalence of gambling problems in the U.S. Military, with around 5% of heavy drinkers reporting 5 or more gambling problems in the DSM-IV criteria (suggestive pathological gambling). In a study of USAF recruits found that GD was increased among those who reported frequent binge drinking (Steenbergh et al., 2008). Similarly, a study of military personnel receiving treatment for GD in the U.S. Military, participants were eight times more likely to have reported prior substance misuse than the general military population (Wilson et al. 2018).

#### Mental health & PTSD

Five sources in the present review (GAO 2017; GSU 2010; Kennedy 2006; Weis and Manos 2007; Wilson et al. 2018) discussed mental health as a wider psychiatric condition, while two sources (GAO 2017, GSU 2010) discussed PTSD. Two studies in this review reported results from measurement of mental health and its association with GD in an active service personnel. Kennedy (2006) found that 9 of 35 individuals receiving treatment for gambling were also screened and subsequently treated for major depressive disorder, while Wilson et al. (2018) found that two thirds of those in treatment for GD in the U.S. military had also reported a prior psychological condition (compared with 13% of the general military population). No studies explicitly measured or related PTSD with GD in active service personnel.

#### Suicide prevention

The association between gambling addiction and increased risk of suicide and suicide ideation is addressed by five sources (Ashley 2017, GSU 2010, GAO 2017, Kennedy 2006 and Weis and Manos 2007). Gambling is acknowledged as an increased risk factor for suicide ideation among veterans (Ashley 2017) and the general population (Weis and Manos 2007, GAO 2017), and it is argued that such relationships are also likely to exist in active duty personnel.

One source (Kennedy 2006) reports that among service personnel undertaking treatment for pathological gambling in a psychiatric centre, 20% (7 of 35) endorsed suicide ideation, with three patients having made suicide attempts. The study also reported that targeted treatment of pathological gambling with suicide prevention was successful in preventing further suicide ideation.

### Secondary diagnosis and combined screening/treatment

Gambling disorders are often diagnosed during treatment for another condition such as depression, financial trouble, relationship/marital issues and substance misuse.^6^ Similarly, the focus on substance misuse and psychiatric help services in a U.S. Military policy review suggest that ‘few seek treatment directly for gambling disorder, and they instead seek treatment for other conditions such as depression (GAO, 2017: p. 18). Four sources argue directly for the inclusion of gambling screening into pre-existing mental and physical health exams (GAO 2017, Kennedy 2006, Kennedy 2006 and Weis and Manos 2007).

## Discussion

The review systematically examined the extent of evidence relating to GD and associated harm within active-duty Anglophonic defence settings. Findings suggest that the evidence base is overwhelmingly constrained to studies of the U.S. military. Prevalence estimates for GD in the military population are available for the U.S. Military (though discontinued since 2003) indicating that GD occurs with roughly similar frequency to the general population, though use of a less sensitive measure of GD (DSM-III & DSM IV instead of the more commonly used SOGS) indicates that comparable prevalence rates may be higher than reported. In addition, concerns on the presence or lack of confidentiality for individuals in the military reporting GD (Kennedy 2005, 2006) suggest that serving personnel may feel less comfortable revealing any issues they may have had during a defence-run health survey.

One area in which the existing evidence is consistently strong is in measuring GD in the context of other behaviours that jeopardise physical and mental health among active duty service personnel (though still only in U.S. contexts). Sources found associations between GD and heavy drinking (Bray 1992, 2002), and violence, drink driving and smoking (Steenbergh et al. 2008). Furthermore, Wilson et al. (2018) found that 67% individuals receiving treatment for GD had prior mental health condition and 47% had prior issues with substances. Therefore, review findings strongly suggest that GD in defence settings is often concentrated in individuals whose underlying mental condition manifests in more than one form of mental distress or problematic behaviour. This is a reiteration of findings from the general population where ‘…the single best gambling-related predictor of dysfunction was not the severity of the disorder, but the severity of the cognitive distortions related to the disorder’ (Shirk et al., 2018).

The association between disordered gambling and PTSD in active duty populations is particularly under studied relative to other military population research. A review by Gates et al., (2012) suggests that rates of PTSD in active duty personnel are roughly twice that of the general population (often comparable with rates reported by veterans) (Gates et al., 2012). A study of Australian veterans by Biddle et al., (2005) described the ‘entrenched gambling culture among PTSD treatment-seeking veterans’ (Biddle, et al., 2005). Westermeyer et al. (2005) and Edens (2012) find that GD is strongly positively correlated with PTSD and positive but not statistically significant correlations were found in Biddle et al., (2005) and O’Toole et al., (1998).

The secondary review objective the review highlighted some of the most salient research opportunities for understanding gambling in military settings. First, comprehensive prevalence studies of active duty defence personal to understand levels of gambling participation and harm in military populations is a high priority. Second, there is a need for dedicated gambling services for active-duty personnel and research is needed to evaluate any programs or services robustly (Ashley 2017). Overall,^7^ evidence of services or the treatment of GD in the military remains extremely limited. Finally, very few qualitative case studies into the unique circumstances surrounding gambling in the military have been conducted, suggesting that detailed understanding of context is missing from the current evidence base.

## Conclusion

This is the first systematic review investigating GD among members in active service in a specific set of defence populations. A significant research gap exists internationally regarding the prevalence of gambling harm among active-duty defence personnel. What is consistently strong (in U.S. studies) is measuring GD in the context of other behaviours that jeopardise physical and mental health among active duty service personnel. However, structural and cultural barriers appear to be a primary reason for military forces and active-duty personnel refraining from recognising or engaging with GD. Although it will be of great interest to see the flow on effects of mandated GD screening in the US military, perhaps in other military settings, screening or prevalence studies should be conducted externally and independently.

## Abbreviations

ANZAC: Australian and New Zealand Army Corps
DoD: Department of Defence
GAO: Government Accountability Office
GD: Gambling disorder
ICT: Internet and communication technologies
PRISMA: Preferred reporting items for systematic reviews and meta-analyses
PTSD: Post-traumatic stress disorder
SOGS: South oaks gambling screen
U.K.: United Kingdom
U.S.: United States

## Footnotes

1 *The Diagnostic and Statistical Manual of Mental Disorders*, *Fifth Edition* defines gambling disorder as “a preoccupation with gambling and a loss of control”.

2 Steps to a scoping review are: (1) identify the research question, (2) identify relevant studies, (3) select relevant studies, (4) chart the data, (5) collate, summarise and report the results.

3 Cochrane Collaboration Glossary, 2010. Available from https://www.cochrane.org/training/cochrane-handbook. Higgins JPT, Green S (editors). Cochrane Handbook for Systematic Reviews of Interventions Version 5.1.0 [updated March 2011]. The Cochrane Collaboration, 2011. Available from www.cochrane-handbook.org. Last JM (editor), A Dictionary of Epidemiology, 4th Ed. New York: Oxford University Press, 2001. Schünemann H, Brożek J, Oxman A, editors. GRADE handbook for grading quality of evidence and strength of recommendation. Version 3.2 [updated March 2009]. The GRADE Working Group, 2009. Available from https://www.cc-ims.net/gradepro.].

4 National Research Council (US) Committee on the Social and Economic Impact of Pathological Gambling. Pathological Gambling: A Critical Review. Washington (DC): National Academies Press (US); 1999. 3, Pathological and Problem Gamblers in the United States. Available from: https://www.ncbi.nlm.nih.gov/books/NBK230631/.

5 There is evidence that the SOGS typically overestimates problem gambling relative to the DSM criteria: Volberg, R.A. 1998. Methodological Issues in Research on Problem Gambling. Paper commissioned by National Research Council, Committee on the Social and Economic Impact of Pathological Gambling, Washington, DC. Gemini Research, Northampton, MA.

6 Carroll, A., Davidson, T., Marsh, D., & Rodgers, B. (2011). Help-seeking and uptake of services amongst people with gambling problems in the ACT. *Canberra: Australian Capital Territory Gambling and Racing Commission*. p. 66.

7 Reports of an ongoing Harm Minimisation scheme in the British Armed Forces since 2016 run by *GambleAware* and EPIC Risk Management are available, though no results or evaluation have yet been made public.
